# Can the digital economy contribute to rural revitalization? A case of from China?

**DOI:** 10.1371/journal.pone.0310313

**Published:** 2024-10-07

**Authors:** Yuanquan Lu, Yuan Meng, Li Chen, Yuan Zhang

**Affiliations:** 1 School of Public Administration, Chongqing University, Chongqing City, China; 2 School of Politics, Law and Management, Yan’an University, Shaanxi City, China; 3 School of Economics and Management, Chongqing Normal University, Chongqing City, China; Zhongnan University of Economics and Law, CHINA

## Abstract

This study establishes a multi-dimensional evaluation index system for measuring digital economy and rural revitalization. It explores the impact of digital economy on China’s rural revitalization and its mechanism using dynamic balanced panel data of 30 provinces and cities in China from 2012 to 2020 and adopting two-way fixed effect and mediated effect models. The results reveal that digital economy promotes rural revitalization in China; however, differences exist in its impact on industrial prosperity, ecological livability, civilized rural customs, effective governance, and affluent living under rural revitalization. Heterogeneity analysis shows that the impact of digital economy on rural revitalization is more pronounced in eastern China, with high levels of new urbanization and areas for big-data experimentation. In addition, this thesis verifies that technological innovation and human capital are two important transmission paths through which digital economy affects rural revitalization in China. The conclusions provide theoretical guidance and practical support for China and other developing countries to vigorously develop their digital economies and comprehensively revitalize the countryside.

## Introduction

**Fig 1:**
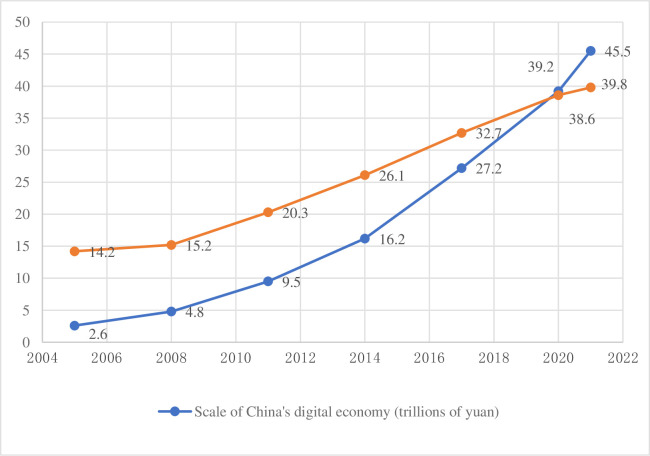
Scale of China’s digital economy and its ratio to GDP, 2005–2021.

The 2030 Agenda for Sustainable Development’s primary goal is to eradicate poverty in all of its manifestations worldwide. As the world’s largest developing country, China has long been committed to solving the problem of poverty. As an important strategy in China, the policy of precise poverty alleviation plays a pivotal role in the eradication of poverty. From 2014 to 2020, China’s precision poverty alleviation policy has achieved remarkable results [1]. Subsequently, China entered the stage of comprehensively revitalizing the countryside. In this stage, along with the continuous improvement of digital infrastructure, the digital economy centered on the Internet and big data technology has strong innovation vitality and growth potential, and has become a new driving force revitalize the countryside and promote our economic development. The China Academy of Information and Communications Technology released the White Paper on Global Digital Economy (2022) (hereinafter referred to as the White Paper). The White Paper shows that the size of China’s digital economy has increased from 2.6 trillion yuan in 2005 to 39.2 trillion yuan in 2021, and its share of GDP has increased from 14.2% to 38.6% (Fig 1).

Constrained by factors such as differences in Internet penetration rates, imbalanced regional economic development, and uncertainty in the external environment, there is a significant gap between the development of China’s digital economy in urban and rural areas. The 2023 White Paper showed that China’s digital economy would account for as much as 50.2% of GDP in 2022, but the penetration rate of the digital economy in agriculture would be only 10.5%. In addition, the Outline of the Fourteenth Five-Year Plan for the National Economic and Social Development of the People’s Republic of China and the Vision 2035 Outline recommend that China “insist on giving priority to the development of agriculture and rural areas, and comprehensively push forward the revitalization of the countryside” and “accelerate digital development, and build a digital China and a digital countryside.” Against this backdrop, this paper must study the impact of the digital economy on China’s rural revitalization and its transmission mechanism. This study places digital economy and rural revitalization under the same analytical framework and explores whether digital economy promotes rural revitalization. If it does, this study considers whether this promotion varies according to the level of economic development and new urbanization and the big data pilot zones set up by the Chinese government. Furthermore, the research attempts to identify the mechanism by which digital economy affects rural revitalization. The research findings can provide academic and empirical support for the government to optimize policies related to the development of digital economy and vigorously promote rural revitalization.

**Fig 2:**
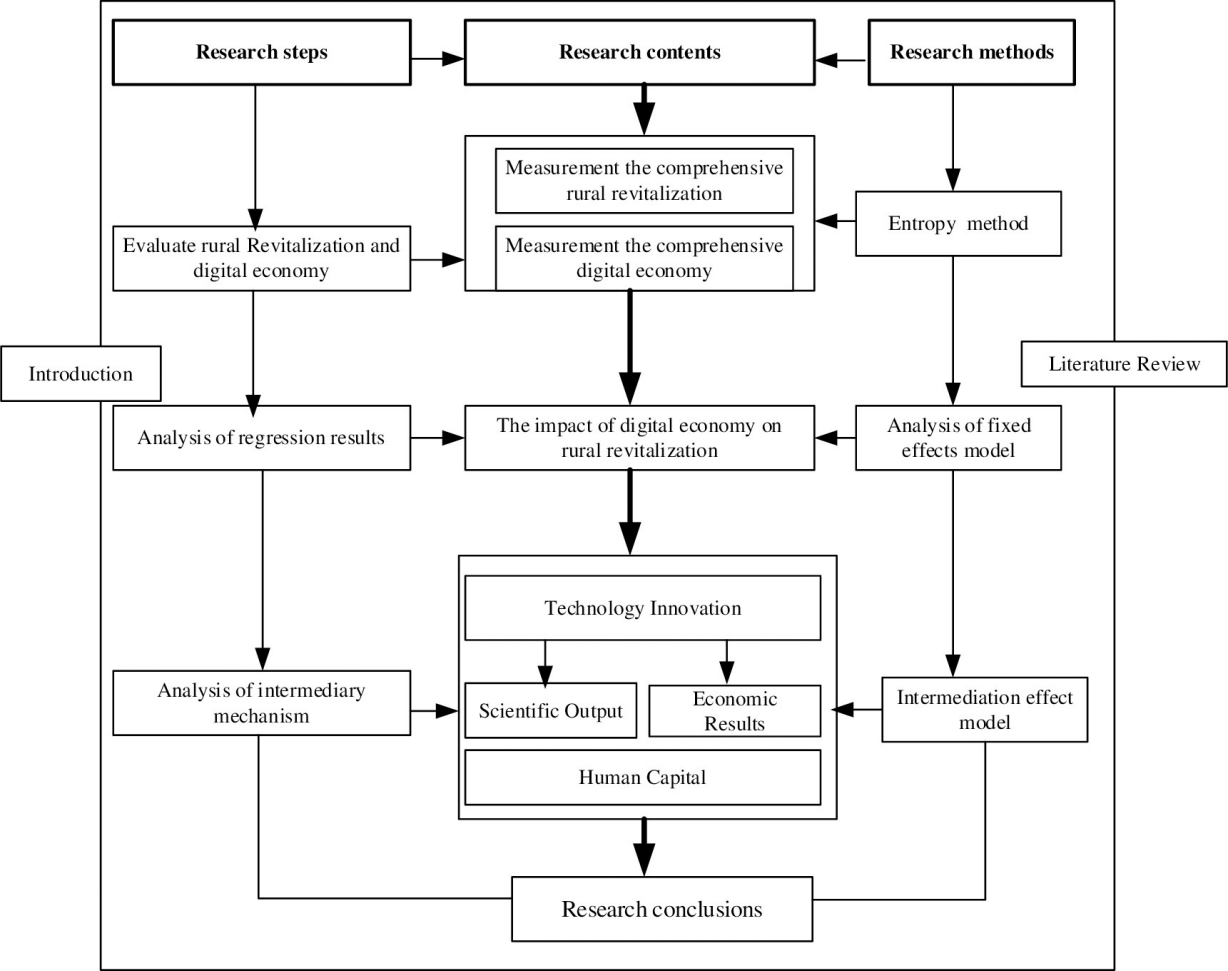
Research framework.

The remainder of the study is structured as follows. The second section presents a literature review, and the third section covers the theoretical analysis and research hypotheses. The fourth section discusses the study, including the data sources, model development, and variables. The fifth section presents the empirical findings, mostly of fixed-effects model analysis, endogenous processing, robustness testing, and heterogeneity analysis. The sixth section examines the mediating effect model, while the seventh one comprises conclusions and policy proposals. The last part is a conclusion and policy recommendations. The research framework diagram for this manuscript is shown below (Fig 2):

## Literature review

### Definition of the digital economy

The digital economy was first discussed in 1996 in the book Digital Economy: Prospects and Crises in the Age of Network Intelligence by Tapscott, which focused on the characteristics of this type of economy [2]; however, no precise definition was provided. Since the advent of the digital economy, related research has progressed through the information economy, Internet economy, and new economy [3–5]. There is no consensus on the definition of digital economy because of disparities in the understanding of its development over time [6, 7]. Several scholars have discussed the more specific and general concepts of digital economy in depth [8, 9], while others have referred to digital economy as economic activities in which goods and services are exchanged digitally [10]. Furthermore, Kotarba has argued that in the digital economy, infrastructure, e-commerce, and e-transactions significantly depend on ICTs [11]. As research has progressed, the digital economy has been considered an economic activity based on digital information (including data elements) as its main resource, the Internet as its main information carrier, digital technology innovation as its driving force, and various new business models as its expression [12]. Scholars generally agree with and endorse this description, which also prevails in this study. Moreover, the platform, sharing, and working economies are examples of the narrow digital economy, whereas e-commerce, Industry 4.0, precision agriculture, and the algorithmic economy are examples of the wide digital economy [8].

### Indicator measurement and other research on the digital economy

Researchers have measured the dimensions of the digital economy from various perspectives; thus, while studies have attempted to develop a system of digital economy indicators at different levels, no consensus exists regarding such a system [13–17]. Furthermore, some research institutions have unveiled their own digital economy evaluation index systems. For example, using the levels of digital industrialization, industrial digitization, and digital governance, the Institute of Information and Communication Research has devised a system for measuring the progress of China’s digital economy [18]. Further, with further research, scholars examined the impact of the digital economy on other aspects, such as employment structure [19], innovation capacity [20], optimization of resource allocation [21], economic growth [22], increase of employment opportunities [23, 24], reduction of carbon emissions [25], and green total factor productivity of the manufacturing industry [26].

### Study on the measurement of rural revitalization, promotion of rural development

The decline of the countryside is a global problem, and all countries have taken measures to promote rural development [27]. However, domestic and foreign scholars have presented differing views on measuring rural development. Foreign scholars have proposed the use of the rurality index to reflect the degree of rural development [28]. Some scholars have used 16 indicators, such as population density, employment rate, and travel mode, to portray the degree of change in the British countryside [29]. In contrast, rural revitalization is a strategy unique to China, implemented to address the decline of the Chinese countryside. Thus, Chinese scholars have constructed a system of indicators to measure rural revitalization from diverse perspectives [30, 31]. In addition, the financial policies promote rural development; furthermore, an increasingly liberal financial policy implies greater contributions to rural economic development [32]. Luo et al. have suggested that the revitalization of the Korean countryside should focus on enhancing innovation in various fields and improving the rural governance mechanism innovatively to meet rural development needs [33]. Another study has argued that positive policy incentives and internal organizational reforms in rural areas could contribute to revitalizing Japan’s rural areas and thus promote the country’s sustainable development [34].

### Digital economy’s effect on rural revitalization

Research on the digital economy’s impact on rural revitalization focuses on certain limited aspects. One study has suggested that in the context of the digital economy, digitalization is indispensable for revitalizing China’s rural industries [35]. Moreover, the digital economy can help achieve the goal of effective rural governance. As a key manifestation of the digital economy, the Internet has simplified administrative procedures, thereby improving rural governance [36]. The deep integration of the digital economy and agricultural development has gradually shaped the digital transformation of agriculture; facilitated agricultural production, industrial upgradation, and the broadening of sales channels; and promoted farmers’ income growth [37]. Furthermore, a study examined the relationship between the digital economy and rural revitalization and found that these two elements are coupled and coordinated [38].

Through a literature review, this manuscript finds that there is a wealth of research on the digital economy and rural revitalization, which lays the foundation for this study. However, previous studies have focused more on "Internet" as the main criterion for measuring the digital economy. In the existing research on the impact of the digital economy on China’s rural revitalization, more attention has been paid to the causal relationship between the two, while ignoring the differences in the different characteristics of this relationship. More importantly, previous literature has not yet paid attention to the internal mechanism of the impact of the digital economy on China’s rural revitalization. This fully demonstrates that there is still room for further expansion of the research on the impact of digital economy on China’s rural revitalization. Accordingly, the main contributions of this manuscript are as follows:

First, various dimensions of the digital economy, such as digital infrastructure, industry digitization, and digital industrialization, are used to develop a digital economy evaluation index system, which adds to and improves extant research. Second, we analyze the differences in the digital economy’s effects on China’s rural revitalization based on the level of new urbanization and the intensity of national policies, contributing to a deeper understanding of the digital economy’s effects. Third, the mediating model accurately verifies the mechanism of the role of science and technology innovation (STI) and human capital in the impact of the digital economy on rural revitalization in China. We provide an overview of the actual situation in rural China and use indicators in rural areas of the country, obtaining significant findings regarding the digital economy and rural revitalization; thus, this study complements and enriches the existing literature.

## Theoretical analysis and research assumptions

### Theoretical analysis of the digital economy’s impact on rural revitalization in China

The basic requirements for rural revitalization include industrial prosperity, ecological sustainability, civilized rural culture, efficient governance, and a high standard of living. The digital economy impacts these five characteristics of rural revival.

First, the digital economy promotes industrial prosperity. On the one hand, digital technology facilitates rural industries’ transition from traditional to modern agriculture. The increasing impact of digital technology propels the transformation and upgradation of labor-intensive conventional agriculture to modern agriculture based on the integration of digitization, intelligence, and precision. Furthermore, sensor technology enhances agricultural production efficiency and strengthens the industry’s ability to prevent external environmental hazards [39], fostering its growth. On the other hand, digital technology has disrupted the rural industrial landscape and pushed the industry in a more progressive direction. The Internet+ approach to developing rural agriculture has resulted in Internet+ modern agriculture, contributing to the growth of rural logistics, tourism, and catering businesses and fostering the development of secondary and tertiary industries, thereby enhancing the industrial structure in rural areas.

Second, the digital economy is environmentally beneficial and influences the ecological viability of rural areas. In developing the digital economy, its ability to combine data and ecological needs contributes to establishing a harmonious ecological environment. Under environmental regulation, the digital economy’s big-data platform enables real-time monitoring of polluting businesses, which enhances the environmental consciousness of businesses and the environmental health of rural areas.

Third, the digital economy contributes to local customs and civilization. Digital platforms for farmers can facilitate access to learning opportunities with the Internet as its conduit. Furthermore, the digital economy plays a significant role in the transition to a new civilization and fosters new norms, harmonious village customs, and the growth of a civilized rural environment.

Fourth, the digital economy can improve the efficiency of rural administration. Five new digital technologies have improved in maturity and usage, namely, the Internet of Things, cloud computing, big data, artificial intelligence, and blockchain [40]. These five technologies enable rural residents to voice their legitimate and reasonable demands and contribute to creating a favorable environment wherein diverse subjects participate in the modernization of the rural governance system. Moreover, digital technology in rural governance increases the business capacity of grassroots governance subjects, promotes an effective connection between the village-level and higher government bodies, increases the transparency of and villagers’ trust in rural governance, and enhances the government’s overall effectiveness.

Fifth, the digital economy contributes to farmers’ wealth. On the one hand, the digital economy can increase rural employment opportunities, allowing rural citizens to enhance their income. On the other hand, rural dwellers can use digital technology to overcome geographical obstacles to a certain extent, particularly those in remote places. Furthermore, digital technology can decrease farmers’ information asymmetry in the market, enhance the efficiency of their economic operations, mitigate the numerous risks they encounter, and boost their likelihood of stable income growth. Therefore, we present the following hypothesis.

Hypothesis 1: The digital economy positively contributes to revitalizing rural China.

### Impact path of the digital economy on China’s rural revitalization

#### The digital economy influences China’s rural revitalization through science and technology innovation

The digital economy can enhance businesses’ technological innovation on three levels by offering them diverse resources and financial support in market operations.

First, the digital economy provides firms with financial support for STI. As a crucial digital economy component, digital-inclusive finance fosters firms’ STI [41]. Furthermore, it combines digitization with conventional approaches. For technological innovation and digital technology empowerment, firms integrate fixed financial activities into the digital space via digital platforms, significantly reducing the access barrier, operation cost, and transaction cost of financial services [42]. This alleviates the difficulties faced by firms excluded from traditional financing and provides financial support to firms for STI activities [43]. Second, the digital economy improves the capabilities of STI firms. As the foundation of the digital economy, digital technology eliminates the constraints of resources such as technology, labor, and capital on economic development and increases the free flow of these resources within China’s provinces, which, to some extent, have reduced the barriers between innovation phases and unified the innovation process [44]. Furthermore, the Internet-based digital economy promotes increasingly effective knowledge exchange and collaboration between businesses, enhances resource allocation efficiency, and contributes to the proliferation and diffusion of knowledge, information, and data, thereby fostering the growth of STI activities and improving innovation capability. Third, the digital economy has stimulated firms’ potential for technological innovation. Digital platforms are essential to the digital economy’s development, providing consumers with a wide range of goods and boosting their demand for high-quality products [45]. This demand drives firms’ technological innovation.

Conversely, STI has contributed to rural revitalization in China. The first effect is industrial structure optimization. On the one hand, STI boosts the optimization of the industrial structure and is a new “factor of production” that integrates new products, markets, and organizations into the production system [46]. By improving labor skills, material and technological bases, and management levels, firms shift from low-yield to high-yield industries; this allows them to form industrial agglomerations, scale effects, and transfer factor resources to the agriculture, industry and servicessystematically, thus optimizing the industrial structure. This optimization also promotes rural revitalization. Moreover, STI promotes the integration of the three major industries. The potential for STI can facilitate the effective integration of industries and promote the selection of industries, cultivation of talents, and risk prevention, especially in the “purchase, storage, marketing, and service” domains for developing rural.

The second effect involves coordination and linkage. Schumpeter’s theory of science and technology innovation provides a theoretical explanation for the digital economy to promote the development of rural revitalization. Schumpeter first explicitly mentioned scientific and technological innovation in the German version of Theory of Economic Development in 1912, and he pointed out that scientific and technological innovation is a necessary factor for the economic development of a country, and that scientific and technological innovation can combine and allocate factors of production such as labor, capital, and land, and thus become a key driving force for economic progress [47]. Coordination and linkage is a systematic and complex process that can only be realized through the comprehensive synergy of its corresponding ecological environment, supporting funds, public services, and relevant policies. Against the backdrop of rural revitalization and STI, rural areas establish functional, spatial, structural, and optimized ecological and value-added technology systems. Furthermore, they adopt industrial development, ecological restoration, energy conservation, and emission reduction technologies, enhancing farmers’ household income and waste treatment in production and life, improving rural residents’ living standards, creating a harmonious production and living environment, and promoting rural public services. Thus, we propose the following hypothesis:

Hypothesis 2: The digital economy revitalizes China’s countryside by enhancing STI.

#### The digital economy drives China’s rural revitalization through human capital

First, as the digital economy continues to grow, it places increasing demands on the human capital of the rural labor force. In the context of the digital economy, many jobs are gradually dispersed, with “data” at the core, giving rise to many new digital industries. This situation has placed significant demands on the human capital of rural workers, for example, in rural e-commerce. For rural workers to adapt to this trend, they must improve their human capital in terms of education and vocational skills through “learning by doing” or “re-education” [48, 49]. In comparison, the digital economy provides opportunities for rural residents to improve their capabilities. With the Internet as its main vehicle, the digital economy is vital in improving human capital. Before the digital age, knowledge and technology were primarily disseminated in rural areas via books and teachers, with limitations of space and time. Thus, ensuring rural residents’ access to high-quality education resources, advanced ideas, and scientific and medical methods was difficult. The arrival of the digital age has transcended the limits of information dissemination, facilitating the spread of knowledge, experience, and technology to rural areas [50]. Rural residents, especially workers, can obtain abundant online learning resources, such as professional skills and health knowledge, through digital platforms. These resources can be accessed anytime and anywhere via the Internet as the primary carrier, enhancing the human capital of rural workers.

Second, human capital has contributed to revitalizing China’s countryside by promoting the development of agriculture, rural areas, and farmers. Schultz’s human capital theory provides a theoretical basis for the digital economy to promote rural revitalization [51]. This theory emphasizes that human capital is the fundamental source of modern economic development. Therefore, human capital, mainly represented by education, is the key element in the rural revitalization strategy. The specifics include the following three primary levels.

1. For agricultural and rural industrial development, education provides dual-factor support for STIs and the labor force. In rural areas, education nurtures professional talents for rural development. In turn, professionals empower agricultural production and management methods through STI, thereby increasing agricultural productivity. Moreover, education can promote the integration of rural tourism, leisure agriculture, and agricultural and sideline product processing to achieve industrial prosperity.
2. Regarding rural development, education, especially rural cultural education, plays a critical role in passing down traditional cultures and promoting the construction of rural civilizations. By conducting cultural knowledge lectures, organizing cultural activities, and cultivating cultural elites [51], a healthy rural cultural environment can be created to achieve the goal of rural civilization construction. Furthermore, grassroots governance subjects with higher education levels possess greater knowledge on scientific governance concepts and stronger grassroots governance abilities, thereby facilitating effective rural governance.
3. Regarding farmers, under the premise of health, education is a primary means for realizing farmers’ human capital and shared prosperity. The human capital farm households acquire through vocational education or skills training contributes most to the income of rural workers [52], helping them earn a stable and sustainable income and thus live comfortably. Accordingly, we propose the following hypothesis.

Hypothesis 3: The digital economy revitalizes China’s countryside by improving rural human capital.

## Study design

### Data source

This study uses panel data from 30 Chinese provinces (excluding Hong Kong, Macau, Taiwan, and Tibet) from 2012 to 2020. This study period was chosen because the center of gravity of China’s economic development was mainly urban until 2012, with little attention paid to rural areas. Since 2012, after the 18th Party Congress, China has paid more attention to rural areas while developing its economy. Therefore, this study takes 2012 as the starting point. The study period ends in 2020 owing to constraints in the availability of post-2020 data related to the novel coronavirus pandemic, time of release of the almanac, and method of data collection. At the research object level, this study mainly studies 30 Chinese provinces and cities, such as Beijing, Tianjin, and Shanghai. We exclude Hong Kong, Macau, Taiwan, and Tibet because the data for these regions are difficult to obtain.

### Model construction

#### Baseline regression model

A benchmark regression model based on panel data is established to test the impact of the digital economy’s development on rural revitalization in China.

$${Rural}_{it}=\alpha_{0}+\alpha_{1}{Digit}_{it}+\alpha_{2}Z_{it}+\mu_{i}+V_{t}+\varepsilon_{it}$$ (1)

where *Rural*_*it*_ and *Digit*_*it*_ represent the level of rural revitalization and the level of the digital economy in province *i* or city *i*, respectively, in year *t*. *Z*_*it*_ denotes a set of control variables affecting *Rural*_*it*_ (*see* *Table* 3), while *μ*_*i*_ and *V*_*t*_ denote individual and time fixed-effects, respectively. *α*_0_, *α*_1_, and *α*_2_ denote the parameters to be estimated. Finally, *ε*_*it*_ denotes the random perturbation term.

#### Quantile regression model

We draw on existing research methods [53] to comprehensively capture the digital economy’s impact on rural revitalization at various levels. We measure this impact at the 0.10, 0.25, 0.50, 0.75, and 0.90 conditional quartiles to construct the following equation:

$${Rural}_{it}={\alpha_{0}}^{\left(P\right)}+{\alpha_{1}}^{\left(P\right)}{Digit}_{it}+{\alpha_{2}}^{\left(P\right)}Z_{it}+{\mu_{i}}^{\left(P\right)}+{V_{t}}^{\left(P\right)}+{\varepsilon_{it}}^{\left(P\right)}.$$ (2)

Some indicators discussed above are not repeated here. The range of values for *P* is [0, 1].

#### Intermediary mechanisms

The following model is established to determine how the digital economy influences the rejuvenation of rural China via STI and human capital. It is based on previous methods [54] and equation (1).


$${Med}_{it}=\beta_{0}+\beta_{1}{Digit}_{it}+\beta_{2}Z_{it}+\mu_{i}+V_{t}+\varepsilon_{it}$$ (3)

$${Rural}_{it}=\gamma_{0}+\gamma_{1}{Digit}_{it}+{\gamma_{2}Med}_{it}+\gamma_{3}Z_{it}+\mu_{i}+V_{t}+\varepsilon_{it}.$$ (4)

In equation (3), *Med*_*it*_ represents intermediary-effects variables, which mainly encompass two major categories: STI and human capital, which is described above and will not be elaborated on here. Eq (3) represents the digital economy’s impact on intermediary-effects variables, and Eq (4) reflects the joint impact of the digital economy and transmission path variables on rural revitalization; these are the coefficients of interest in this study. If *α*_1_、*β*_1_、*γ*_1_ are significant but *γ*_2_ is not, STI is the only way for the digital economy to influence rural revitalization in China. Conversely, if *α*_1_, *β*_1_, *γ*_1_, and *γ*_2_ are all significant, STI is a path for the digital economy to influence rural revitalization in China; however, it is not the only path. The other variables are the same as those in Eq (1).

### Variables

#### Dependent variables

The dependent variable is rural revitalization. The research object is rural revitalization at the provincial level. Therefore, based on the existing literature [55] and available data, this study establishes a multi-dimensional rural revitalization indicator system (Rural) comprising industrial prosperity, ecological livability, civilized rural style, effective government, and prosperous living (see Table 1). Regarding the indicators in Table 1, we applied the following treatments. Because of the various levels of indicators, all the above variables are standardized. The entropy value method (Appendix A in S1 File) was used to calculate the weights of the variables discussed in the previous paper [56]. Furthermore, the linear weighted summation method was used to calculate the comprehensive index of rural revitalization for each province and city every year. Finally, the comprehensive index measures rural revitalization, and its value ranges from 0 to 1.

**Table 1:** The rural revitalization index system, definitions, and average weights.

Target Index	Secondary indicators	Three-level indicators	Indicator Definition	Average weight	Attributes
rural revitalization	Industrial prosperity (0.1551)	Unit acres of agricultural machinery power	Total power of agricultural machinery / effective arable land area	0.0371	+
		Agricultural labor productivity (yuan/person)	Value added of primary industry/number of employees	0.0239	+
		Rural land productivity (yuan/mu)	Value added of primary industry / arable land area	0.0439	+
		Per capita output value of agriculture, forestry, animal husbandry, and fishery (yuan)		0.0270	+
		The proportion of the added value of primary industry (%)	Value added of primary industry/regional GDP	0.0232	+
	Ecological Livability (0.2711)	Rural greening coverage rate (%)		0.0374	+
		Park green space per capita in rural areas (square meters)		0.0606	+
		Rural water supply penetration rate (%)		0.0169	+
		Rural sanitary toilet penetration rate (%)		0.0170	+
		Township sewage treatment house penetration rate (%)	Number of township sewage treatment/number of townships	0.0711	+
		Health technicians per 1,000 population in rural areas (persons)		0.0320	+
		Road area per capita in the countryside (square meters)		0.0361	+
	Countryside Civilization (0.1569)	Township cultural station coverage rate (%)	Number of cultural stations in townships/number of townships	0.0178	+
		The proportion of rural full-time teachers at the junior high school level (%)	Full-time rural teachers at the junior high school level / Full-time junior high school teachers throughout the province	0.0241	+
		Rural per capita consumption expenditure on culture and entertainment (%)		0.0255	+
		Public library collections per capita in rural areas (volumes)	Public book collection / rural resident population	0.0505	+
		The average number of secondary school students per rural teacher (students)	Number of elementary school students in rural areas / Number of elementary school teachers in rural areas	0.0390	+
	Effective Governance (0.2341)	Villagers’ participation in the current voting rate (%)	Number of people who voted/number of registered voters in the current period	0.0409	+
		The proportion of village chief "secretary" shoulders (%)	Number of chiefs and secretaries "on one shoulder"/number of village committees	0.0441	+
		The annual average standard of rural minimum living security (yuan/person)		0.0653	+
		Number of urban and rural residents’ social pension insurance participants (Ten thousand people)		0.0355	+
		The proportion of administrative villages that have prepared village plans (%)	Administrative villages that have prepared village plans/all administrative villages	0.0199	+
		The proportion of administrative villages that have carried out village improvement (%)	The number of administrative villages to carry out village improvement / the number of all administrative villages	0.0284	+
	Living well (0.1827)	Per capita disposable income of rural residents (yuan)		0.0315	+
		Ratio of per capita disposable income of urban and rural residents	Per capita disposable income of urban residents / per capita disposable income of rural residents	0.0410	-
		Per capita consumption level of rural residents (yuan)		0.0382	+
		Engel coefficient of rural residents (%)	Year-end household expenditure on food, tobacco, and alcohol / Total year-end household expenditure	0.0721	-

**Fig 3:**
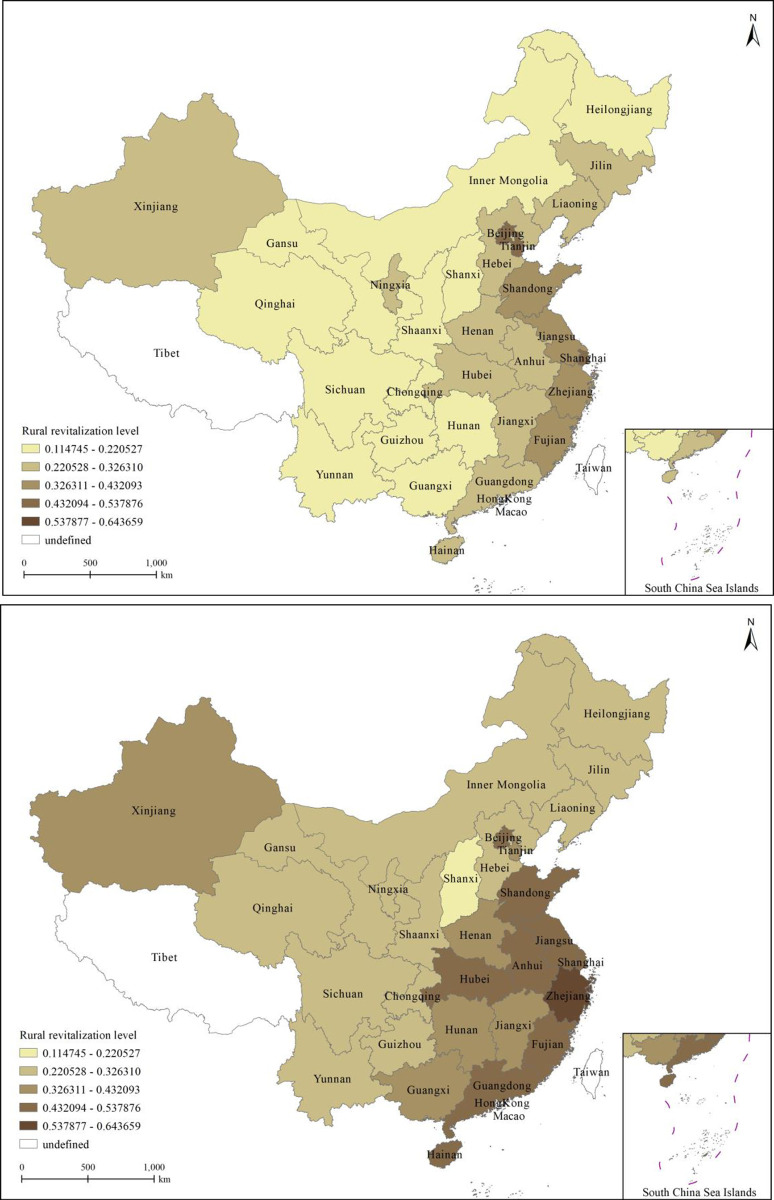
(a) Rural revitalization level in 2012. (b) Rural revitalization level in 2020.

Fig 3(A) and 3(B) reflect the geographical distribution of the level of rural revitalization in 30 Chinese provinces and cities from 2012 to 2020. These two graphs show that rural revitalization in China significantly improved from 2012 to 2020; however, interprovincial variability emerges. Fig 3(A) shows that most Chinese provinces and cities (except Beijing, Tianjin, and Shanghai) were in the first echelon of rural revitalization levels in 2012, i.e., at a lower level. In 2020, most Chinese provinces and cities jumped from the first to the second echelon (and beyond).

#### Independent variables

The digital economy is an independent variable representing the rural digital economy; from this point forward, the rural digital economy will be called the digital economy. While the digital economy is intended for bringing China’s urban and rural areas closer, significant differences exist in developing cities and villages in China. Moreover, considering cities and villages as a whole and assessing the holistic impact of digital economy on China’s rural revitalization can somewhat bias the results. Domestic and foreign scholars have yet to agree on the definition of the digital economy. Drawing on existing research results [57], this study establishes a comprehensive digital economy index (Digit) in terms of digital infrastructure, industrial digitization and digital industrialization (see Table 2). Before the entropy method is applied, all variables in Table 2 are standardized. Based on this, a comprehensive digital economy index is calculated using the entropy value method (Appendix A in S1 File). The value range of this index is [0, 1]; the larger the value, the more developed is the digital economy.

**Table 2:** The digital economy index system, definitions, and average weights.

Target index	Secondary indicators	Three levels of indicators	Indicator Definition	Average weight
Digital Economy	Digital Infrastructure	The proportion of administrative villages with Internet broadband service (%)	Administrative villages with Internet broadband service/all administrative villages	0.0149
		The proportion of rural Internet broadband to households (%)	Rural broadband access users/Internet broadband access users	0.0213
		Cell phone penetration rate (units per 100 people)		0.0408
		Rural Internet penetration rate (%)	Rural Internet users/residents of the rural population	0.0396
	Industry Digitization	Rural e-commerce sales (billion yuan)		0.0913
		Rural e-commerce procurement volume (billion yuan)		0.1015
		Total postal services per capita (yuan)	Total postal business/resident population	0.1073
		Per capita express business volume (yuan)	Express business volume / resident population	0.1419
		Express business revenue by province (RMB million)		0.1165
	Digital Industrialization	Digital Financial Inclusion Index for Rural China (Guo, Feng, et al., 2020) [43]	Peking University Financial Centre	0.0386
		Average service personnel per business branch in rural areas		0.0863
		"Taobao village" number	Research Report on Taobao Villages in China	0.2000

**Fig 4:**
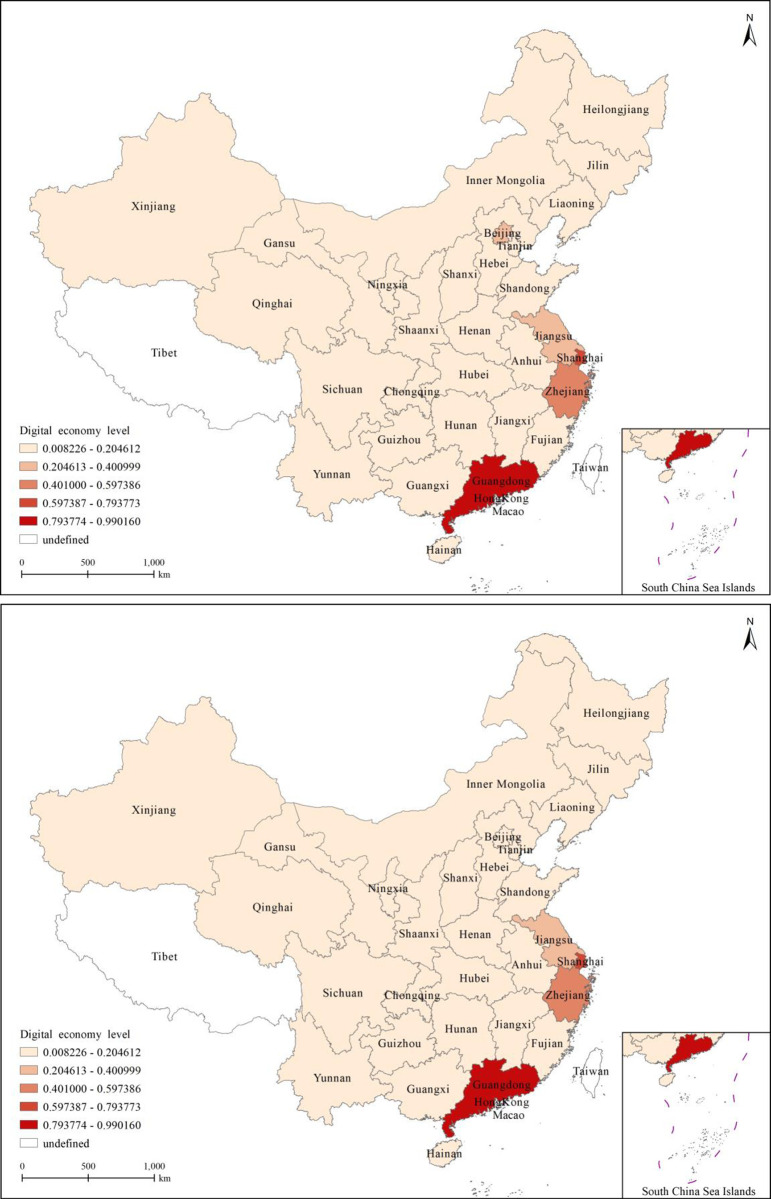
(a) The digital economy level in 2012. (b) The digital economy level in 2012.

Fig 4(A) and 4(B) depict the geographical distribution of digital economy levels in 30 provinces and cities in China in 2012 and 2020, showing significant improvements. In 2012, the level of rural revitalization in most Chinese provinces and cities was at its lowest. In 2020, digital economy levels increased in most provinces and cities; however, most were still in the first tier.

#### Control variables

The following critical control variables were found to possibly influence the expansion of rural revitalization. (1) Regional industrial structure (Industry): In this study, the regional industrial structure is characterized by the ratio of the tertiary industry’s production value to the secondary sector’s output value. (2) Rural financial development (Finance): Rural (county and sub-county) loans are used to assess the evolution of rural financing because of the availability of data. (3) The intervention amount of government support for agriculture (Intervention): Government spending on agriculture, forestry, and water conservation is used to determine the level of government participation in agriculture. (4) Rural population structure (People): The rural population is the cornerstone of rural revitalization, and the composition of the rural population influences the achievement of rural revitalization goals. This study uses the sum of the rural child dependency ratio and the senior dependency ratio as significant variables to characterize the rural population’s structure. (5) The total value of fixed-asset investments in rural areas (Fixed) is an important indicator of development. (6) Rural talents (Trainers). The number of rural workers participating in training is a proxy variable for the rural talent guarantee. (7) Level of urban growth (Urban) is a multi-dimensional evaluation index system for new urbanization constructed based on secondary indicators for economic, demographic, social, ecological, and cultural urbanization. This study normalizes the above variables, and their weights are derived using the entropy method (Appendix B in S1 File); a comprehensive index of new urbanization is then computed. This indicator ranges from 0 to 1.

#### Intermediary variables

(1) STI: When measuring the effect of STI, the STI output is representative of convincing. Based on the available data, two indicators were mainly adopted to measure STI output: the number of patent applications for new agricultural plant varieties and the number of agricultural technology market transactions. (2) Human capital: The rural labor force’s actual human capital per capita was used to denote the rural human capital status (see Table 3).

**Table 3:** Descriptive statistics results of the main variables.

Variable Name	Variable Code	Variable definition and data handling	Minimum	Maximum	Average value	Standard deviation
rural revitalization	rural	Table 1	0.1029	0.9519	0.3665	0.1538
Industrial prosperity	Prosperous	Table 1	0.0019	0.3380	0.0819	0.0662
Ecological Livability	Livability	Table 1	0.0615	0.9810	0.3141	0.1889
Countryside Civilization	Civilization	Table 1	0.1082	0.6660	0.3403	0.1149
Effective Governance	Effective	Table 1	0.1018	0.7050	0.3151	0.1350
Living well	Well	Table 1	0.0136	0.9157	0.4613	0.1993
Digital Economy	Digit	Table 2	0.0348	0.7113	0.1802	0.1697
Regional Industry Structure	Industry	Tertiary industry output value / Secondary industry output value	0.1584	0.5769	1.2507	0.7069
Rural Financial Development	Finance	Rural (county and sub-county) loans (billion yuan), taking logarithms	5.6108	10.5720	8.4938	1.0279
Degree of government intervention in support of agriculture	Intervention	Financial expenditures in agriculture, forestry and water conservancy affairs and other areas (billion yuan), taking logarithms	4.6149	7.2000	6.2186	0.5507
Rural population structure	People	Sum of rural child dependency ratio and elderly dependency ratio (%),	0.4324	0.8747	3.6139	0.1908
Rural fixed asset investment	Fixed	Completed investment in fixed assets in rural households (billion yuan), taking logarithm	1.0986	6.8739	5.3933	1.1344
Rural Talent	Trainers	Number of rural workers participating in training (persons), taking logarithm	4.5539	12.6871	10.2734	1.6361
New urbanization	Urban	Appendix B in S1 File	0.1547	0.8820	0.3583	0.1591
Scientific Output	Patent	Number of patent applications for new varieties of agricultural plants (pieces), taking logarithms	0	6.6307	4.0005	1.3339
Economic Results	Innovation	Agricultural technology market turnover (million yuan), taking logarithm	7.6561	14.8453	12.0097	1.4820
Human Capital	Human	Real human capital per rural labor force ($1000), taking logarithm	3.8862	6.0133	4.7028	0.3855

## Analysis of the empirical results

### Analysis of the regression results

#### Digital economy’s impact on China’s rural revitalization

The Hausman test results suggested that the fixed-effects model is preferable to the ordinary least squares and random-effects models, strongly refuting the original theory; therefore, the fixed-effects model was selected. Table 4 presents the results of a linear estimation of the digital economy’s influence on rural revitalization. Model (1) demonstrates the empirical result without any control variables, and the estimated coefficient of the digital economy is 0.3630, which is statistically significant at the 1% level, indicating that the digital economy contributes to rural rehabilitation. Furthermore, Model (2) integrates the primary control variables that influence rural revitalization in China based on Model (1), indicating a decrease in the estimated coefficients of the digital economy. However, the sign and significance level of the coefficients do not change much, which is consistent with previous findings and confirms Hypothesis 1. This conclusion offers a theoretical basis for China to accelerate the digital economy’s growth to provide new momentum for developing rural revitalization. Considering the widespread adoption of information technologies such as big data, cloud computing, and artificial intelligence, China’s digital economy has made significant strides in scale, growth rate, and application, creating new opportunities to resolve various rural development challenges.

**Table 4:** Analysis of the impact of digital economy on rural revitalization and its specific dimensions.

Variables	(1)	(2)	(3)	(4)	(5)	(6)	(7)
	Rural	Rural	Prosperous	Livability	Civilization	Effective	Well
Digit	0.3630*** (0.0418)	0.1873*** (0.0656)	0.1080 (0.0304)	0.5362*** (0.0635)	0.1360*** (0.0454)	0.2583*** (0.0496)	0.4110*** (0.0781)
Industry		0.0130 (0.0118)	0.0007 (0.0055)	0.0090 (0.0114)	0.0091 (0.0081)	0.0054 (0.0089)	0.0271* (0.0140)
Finance		0.0354** (0.0153)	0.0149** (0.0071)	0.0811*** (0.0149)	0.0415*** (0.0106)	0.0458*** (0.0116)	0.0359* (0.0183)
Intervention		0.0100 (0.0291)	0.0426*** (0.0135)	0.0366 (0.0126)	0.0367* (0.0202)	0.0323 (0.0220)	0.0163 (0.0347)
People		-0.1482** (0.0784)	-0.1230** (0.0501)	-0.0776*** (0.0126)	-0.0307*** (0.0093)	-0.1475* (0.0769)	-0.0020** (0.0010)
Fixed		-0.0274** (0.0131)	-0.0345*** (0.0061)	-0.0804*** (0.0126)	-0.0290*** (0.0904)	-0.0327*** (0.0099)	-0.0558*** (0.0155)
Trainers		0.0009 (0.0065)	0.0019 (0.0030)	0.0002 (0.0063)	0.0038 (0.0045)	0.0036 (0.0049)	0.0094 (0.0077)
Urban		0.3302*** (0.0580)	0.1271*** (0.0484)	0.3260*** (0.1003)	0.2950*** (0.0629)	0.4113*** (0.0757)	0.8388*** (0.1142)
Constant	0.301* (0.0103)	0.202* (0.0105)	0.232*** (0.0488)	0.174* (0.102)	0.308*** (0.0727)	0.176** (0.0795)	0.285** (0.125)
Province FE	NO	YES	YES	YES	YES	YES	YES
Year FE	NO	YES	YES	YES	YES	YES	YES
Panel model setting F-value	75.41*** [0.0000]	39.88*** [0.0000]	18.67*** [0.0000]	50.35*** [0.0000]	5.02*** [0.0000]	44.36*** [0.0000]	38.89*** [0.0000]
R^2^	0.169	0.292	0.384	0.628	0.146	0.568	0.492
Observation	270						

Note: ***, **, * denote significant at 1%, 5%, and 10% statistical levels, respectively, () data are standard errors, p-values in [].

Column (2) of Table 4 contains control variables, showing a nonsignificant positive relationship between regional industrial structure and rural revitalization. This finding suggests that regional industries can promote rural revitalization in China but not at a statistically significant level as the regional industrial structure can promote the development of industries in China’s rural revitalization and realize the prosperity of rural industries. Meanwhile, rural revitalization in this country comprises five dimensions, namely, prosperous industry, ecological livability, civilized countryside, effective governance, and living conditions. Only the combined efforts of these five components can promote Chinese rural revitalization. The estimated coefficient of the scale of rural finance is significantly positive, suggesting that the construction of a high-level capital market is essential for promoting the development of rural revitalization. Furthermore, the coefficient of the degree of government intervention in rural areas is positive but statistically insignificant. Government intervention in agriculture promotes rural revitalization development, especially rural agricultural development; however, the degree of this intervention remains uncertain owing to multiple factors, such as the market environment, business conditions, transportation conditions, and various disasters, preventing government intervention in agriculture from playing its proper role. Rural population structures and village fixed-asset investment completion impede rural revitalization and were significant at the 1% and 5% statistical levels. The main reason for the former is that prominent child and elderly dependency ratios indicate inadequate labor support for rural development and revitalization. Village fixed-asset investment completion occurs because most rural households prefer investing in items such as houses and cars; these items are not necessary for production and cannot therefore positively influence the development of rural revitalization. The estimated coefficient of rural talent is positive but insignificant. On the one hand, rural workers participate in various vocational skills training programs offered by the government and different firms; however, they are more likely to be taught the theoretical aspects and may lack practical experience in using these skills. Hence, in addition to their generally low education, they cannot achieve the expected effect. On the other hand, the high mobility of the rural youth decreases the labor force available in rural areas, leading to insufficient talent resources to support local industrial development. The effect of new urbanization on rural revitalization is significantly positive, indicating that it encourages rural revitalization by promoting the free flow of urban and rural elements. This situation offers full play to the advantages of resource endowments in urban and rural areas; guides the transfer of capital, talents, and technology to rural areas; accelerates the deep integration of industries and elements between urban and rural areas; and lays the foundation for the development of rural revitalization.

#### The digital economy’s impact on rural revitalization in various dimensions

Models 3–7 in Table 4 reveal that the coefficients of the digital economy on rural revitalization in the five dimensions of flourishing industry, ecological livability, civilized countryside, effective governance, and affluent life are 0.1080, 0.5362, 0.1360, 0.2583, and 0.4110, respectively. This finding indicates that the digital economy enhances rural revitalization in all five dimensions, with the largest effect on ecological livability, whereas the effect of industrial prosperity is small and not statistically significant. The specific reasons are as follows. First, the digital economy is environmentally friendly; hence, the growth of this type of economy will not lead to environmental pollution. Second, with the Internet as its main carrier, digital technology allows numerous people, including the public, the government, and social organizations, to monitor rural businesses’ sewage systems. Nonetheless, to achieve a digital economy and promote industry prosperity, which is a long, dynamic, and complex process, we must discuss financial investment, human resources support, technological innovation, various industrial development areas, and the digital economy’s renewal.

### Endogenous processing

Although this study found that the digital economy promotes rural revitalization, this finding may be endogenously biased. Therefore, based on previous research results [58], an endogeneity test is conducted. The instrumental variable of the digital economy index of each province and city this year is taken as the product of the number of national Internet users in the previous year and the historical data of post and telecommunications of each province and city in 1984. On the one hand, regions with greater historical telephone penetration are likely to possess more Internet information technology, and the development of local Internet technology and residential Internet use influences the development of the digital economy, thus satisfying the correlation. On the other hand, the impact of historical telephone penetration on rural revitalization gradually disappears as the frequency of telephone use decreases, thus satisfying the exclusion. The results in Columns (1) and (2) of Table 5 reveal that the digital economy’s effect on rural revitalization development persists with full consideration of the endogenous problem. The results are significant at the 1% level, indicating that after considering the endogenous problem, this result still supports Hypothesis 1.

**Table 5:** Endogeneity treatment and robustness tests.

Variables	(1)	(2)	(3)	(4)	(5)	(6)	(7)	(8)	(9)	(10)
	Phase One	Phase Second	Lags one period	OLS	QR_10	QR_25	QR_50	QR_75	QR_90	Adjusting the study sample
IV-Digit	0.5183*** (0.2124)									
Digit		0.1881*** (0.0667)	0.1923*** (0.0718)	0.1871*** (0.0656)	0.1603** (0.0737)	0.2240*** (0.0755)	0.2712*** (0.0707)	0.3704*** (0.0640)	0.3971*** (0.0534)	0.1714*** (0.0402)
Digit _(-1)_			0.4276** (0.2228)							
Control variables	YES	YES	YES	YES	YES	YES	YES	YES	YES	YES
Constant	0.174* (0.113)	0.339* (0.1950)	0.695 (0.5163)	0.107 (0.0997)	0.154 (0.112)	0.163 (0.115)	0.185* (0.107)	0.208** (0.0973)	0.159* (0.0812)	0.107** (0.0369)
Province FE	YES	YES	YES	YES	YES	YES	YES	YES	YES	YES
Year FE	YES	YES	YES	YES	YES	YES	YES	YES	YES	YES
Observation	252	252	240	270	270	270	270	270	270	234
Panel model setting F-value	15.19	46.23	13.36*** [0.0000]							34.57*** [0.0000]

Note: ***, **, * denote significant at 1%, 5%, and 10% statistical levels, respectively, () data are standard errors, p-values in.

### Robustness test

#### Replacing the dependent variable

For regression analysis, one-period-lagged data on rural revitalization were used. Data from the current period cannot help determine the order of the effects of the digital economy and rural revitalization, and there is a high chance of reverse causality. The data presented in Column 3 of Table 5 indicate that the estimated coefficients and importance of the digital economy have not changed much. Compared with the baseline regression findings (Column 2 of Table 4), the digital economy’s lagging effect on rural revitalization is much more significant than the baseline regression results. After adjusting for the possibility of reverse causation, this explains why the digital economy’s effect on rural revival development is significant. This finding is reliable and supports Hypothesis 1.

The reason for the sample size of 252 here is that, except for Tibet, there were only 28 provinces and cities in China where telephones were widespread across all regions in 1984, missing Chongqing and Hainan provinces. In the 1984 administrative divisions of China, Chongqing and Hainan provinces were part of Sichuan and Guangdong provinces, respectively.

#### Quantile regression

Quantile regression was used in this study to determine how the digital economy affects rural revitalization at various quantiles. From the 10th to the 90th quantile of rural revitalization, the estimated coefficients of the digital economy increase and become significant at the 5% and 1% levels, as shown in Columns 4–9 of Table 5. This finding implies the digital economy’s estimated coefficient increases as the rural regeneration quantile increases, thus supporting the first hypothesis.

#### Adjusting the study sample

Typically, Chinese cities and towns are directly subordinate to the central government’s policies and financial support. This results in the development of rural revitalization in municipalities directly under the central government, which have a particular significance. Therefore, data from the four municipalities of Beijing, Shanghai, Tianjin, and Chongqing were excluded for empirical evidence to conclude that the digital economy revitalizes rural areas in China. Column 10 of Table 5 shows that the estimated coefficient of the digital economy is 0.1714, which is statistically significant at the 1% level. Compared with the estimated coefficient in Column 2 of Table 4 (0.1873), the estimated coefficient of the digital economy in Column 10 is lower; however, the coefficient’s sign and degree of significance do not change significantly. Thus, this finding supports Hypothesis 1.

### Heterogeneity analysis

#### Geographical location

Columns (1)–(3) of Table 6 show that the digital economy affects China’s western, central, and eastern regions. The impact coefficients of rural revitalization of 0.1554, 0.1703, and 0.1921, respectively, are all statistically significant at the 1% level. This finding fully illustrates the significant geographical differences in the digital economy’s promotion effect, with the largest effect on the eastern region and the smallest effect on the western. On the one hand, China’s eastern region has a higher economic development, more advanced technology, better infrastructure, and a more open institutional environment. These advantages have laid a solid hardware foundation and software support for the development of the digital economy, enabling it to transform the benefits of data in the digital economy into economic development. On the other hand, compared with the central and western regions, the country’s eastern region can fully utilize the technological advantages of the digital economy. These benefits can be expanded and deepened; thus, the digital economy in this region can spread to and drive the economic growth of the local rural areas, thereby facilitating rural revitalization. In contrast, the western region is relatively lagging in economic development and has a weak digital economy infrastructure, making it difficult to empower the villages fully with digital technology in the digital economy and ultimately weakening the digital economy’s positive effect on rural revitalization.

**Table 6:** Impact of digital economy on rural revitalization under different characteristics of economic development and new urbanization level.

Variables	(1)	(2)	(3)	(4)	(5)	(6)	(7)
	West Region	Central Region	East Region	Low-level New Urbanization	High level of new urbanization	Test area	Non-test area
Digit	0.1554*** (0.358)	0.1703*** (0.3719)	0.1921** (0.4086)	0.1445* (0.0098)	0.1659** (0.1010)	0.4193*** (0.0513)	0.2802** (0.0359)
Control variables	YES	YES	YES	YES	YES	YES	YES
Constant	0.327*** (0.143)	0.368 (0.308)	0.392** (0.165)	0.243** (0.1094)	0.259** (0.1137)	0.238*** (0.1069)	0.159* (0.0874)
Province FE	YES	YES	YES	YES	YES	YES	YES
Year FE	YES	YES	YES	YES	YES	YES	YES
Panel model setting F-value	5.47*** [0.0000]	6.83*** [0.0000]	7.52*** [0.0000]	8.73*** [0.0000]	9.14*** [0.0000]	74.94*** [0.0000]	53.86*** [0.0000]
R^2^	0.146	0.181	0.189	0.182	0.194	0.345	0.297
Observation	99	72	99	172	98	90	180

Note: ***, **, * denote significant at 1%, 5%, and 10% statistical levels, respectively, () data are standard errors, p-values in.

During the sample research period, the following Chinese provinces and cities were designated as Big Data Comprehensive Pilot Zones: China’s first comprehensive pilot zone for big data was established in Guizhou in September 2015; by September 2016, China had two major cross-regional types of comprehensive pilot zones (Beijing-Tianjin-Hebei and Pearl River Delta), four regional divisional-type comprehensive pilot zones (Shanghai, Henan, Chongqing, and Shenyang), and one data infrastructure-integrated development-type comprehensive pilot zone (Inner Mongolia), with the goal of promoting the development of big data.

#### New urbanization level

In 2012, the 18th CPC National Congress formally clarified the need to take the new urbanization “people-oriented” road with Chinese characteristics for the first time. In 2013, the Central Working Conference on Cities and Towns discussed the basic principles and key tasks of promoting this new urbanization, highlighting that the “newness” of new urbanization should be embodied in several aspects, including promoting the urbanization of the agricultural transfer population with a focus on human beings, elucidating the rational and scientific layout of urbanization, improving the efficiency of resource utilization, and attaching importance to ecological and environmental protection. In 2014, the Central Committee of the Communist Party of China and the State Council issued the National New Urbanization Plan (2014–2020), which mentioned that new urbanization is characterized by “people-oriented synchronization of the four urbanizations, optimization of the layout, ecological civilization, and cultural inheritance.” The Twentieth CPC National Congress of 2022 report proposed that new urbanization should be firmly implemented, centered on the citizens and guided by the concept of coordinated, innovative, shared, open, and green development. The above meetings indicate that new urbanization in the Chinese context has three characteristics. First, it places people at its core; people are the city’s main body and the ultimate targets of urbanization. Whether urbanization is aimed at achieving economic growth or social development, it can truly be realized only with people’s involvement. Second, the country should focus on integrating urban and rural development. Furthermore, the government should promote the coordinated development of towns and villages and give full play to the pulling effect of the faster-developing towns and villages to realize the development strategy of “promoting the countryside with the towns.” Third, in the revitalization process, new urbanization should consider governance and environmental protection, strengthen the construction and maintenance of the ecological environment, and adopt a green, low-carbon, and sustainable development approach. In other words, new urbanization comprises the urbanization of people, society, economy, environment, and space. Thus, it can be divided into economic, population, social, ecological, and cultural aspects (See Appendix B in S1 File).

This manuscript uses the mean value of the composite index of new urbanization level (0.3583) as the dividing criterion (See Table 3), and defines those higher than the mean value of the composite index of new urbanization level as high level of new urbanization, and vice versa as low level of urbanization. The regression results shown in Columns 4–5 of Table 6 demonstrate a significant difference in the effect of the promotion of the digital economy on the rural revitalization of provinces and cities at various levels of new urbanization. The effect on a high level of new urbanization is prominent because the digital economy has a superpositioning and multiplier effect, offering new advantages and generating new momentum for new economic, demographic, social, ecological, and cultural urbanization. Compared with low-level new urbanization, high-level new urbanization has a significant radiating effect on the surrounding areas. It accelerates the transfer of capital, technology, information, and talents to rural areas, compensating for rural areas’ shortcomings in industrial development, social governance, and environmental improvement, thereby promoting the revitalization of rural development.

#### Policy implementation intensity

After the agricultural and industrial economies comes the digital economy. This type of economy depends extensively on policy help and strategic planning, which is most apparent in building big-data pilot zones. The Chinese government released the “Action Plan for Promoting Big Data” in 2015, which states that “all provinces and localities in China must accelerate the growth of the big-data industry.” Various provinces and cities in China have formed “Big Data Comprehensive Pilot Zones.” This study distinguishes between provinces that have and have not developed large data centers as “pilot zones” and “nonpilot zones,” respectively, over the sample study period. Columns 6 and 7 of Table 6 reveal that the digital economy’s contribution to the rejuvenation of rural China’s experimental zones is greater and more substantial at the 1% level than in nonexperimental zones. As comprehensive big-data zones were established gradually in China, certain regions in the country have applied data in numerous areas, including industrial development, rural governance, ecological and environmental protection, and livelihood improvement in rural areas. These applications boost rural development and rural revitalization in China.

## The intermediary effect

The results of Eqs (1), (3), and (4) indicate whether the digital economy affects the revitalization of rural areas through STI and human capital. Table 7 presents the entire regression analysis. Column 1 of the intermediary-effects analysis uses the baseline regression result from Column 2 of Table 4 as a reference. Column 2 of Table 7 depicts the digital economy’s influence on scientific research output in STI. The regression coefficient is significantly positive at the 1% significance level, indicating that the growth of the digital economy increases scientific research output. Furthermore, the digital economy and scientific research output in STI have significant regression coefficients in Column 3. In contrast, the digital economy’s direct influence (0.1873 in Column 1) is marginally greater than the coefficient of the combined effect (0.1761 in Column 3), signifying that producing research is an avenue through which the digital economy influences rural revitalization in China. In agreement with this inference, Column 4 shows a significant positive connection between the digital economy and economic outcomes in STIs. Moreover, Column 5 presents the digital economy’s effects on rural revitalization and economic outcomes. The results indicate that the estimated coefficients of the digital economy and economic benefits are 0.1601 and 0.0342 and are significant at 1% and 5% levels, respectively. These findings indicate that the digital economy significantly influences rural revitalization via economic benefits. These results support Hypothesis 2.

**Table 7:** Regression results analysis of the transmission pathway of the digital economy affecting the revitalization of rural China.

Variables	(1) Rural	(2) Patent	(3) Rural	(4) Innovation	(5) Rural	(6) Human	(7) Rural
Digit	0.1873*** (0.0656)	0.4032*** (0.0956)	0.1761*** (0.0650)	0.6443*** (0.0656)	0.1601*** (0.0659)	0.8793*** (0.1205)	0.1692*** (0.0720)
Patent			0.0403** (0.0159)				
Innovation					0.0342** (0.0156)		
Human							0.0836** (0.0341)
Control variables	YES	YES	YES	YES	YES	YES	YES
Constant	0.202* (0.0105)	0.057** (0.113)	0.268** (0.1187)	5.4938*** (1.1187)	2.111** (0.1069)	3.6385** (0.1925)	2.4792** (0.1136)
Area and year control	YES	YES	YES	YES	YES	YES	YES
Panel model setting F value / F value	39.88*** [0.0000]	58.49*** [0.0000]	61.17*** [0.0000]	23.20*** [0.0000]	10.65*** [0.0000]	80.76*** [0.0000]	13.15*** [0.0000]
R^2^	0.292	0.7010	0.2633	0.4600	0.1624	0.4838	0.2594
Observation	270	270	270	270	270	270	270

Note: ***, **, * denote significant at 1%, 5%, and 10% statistical levels, respectively, () data are standard errors, p-values in.

Column 6 demonstrates how the digital economy affects human capital. The results reveal that the projected coefficient of the digital economy is extremely positive, indicating that the digital economy enhances human capital. In Column 7, the regression coefficients for the digital economy and human capital are 0.1692 and 0.0836, respectively. These results are statistically significant at the 1% and 5% levels, and the digital economy’s direct-effect coefficient is larger than the combined-benefit coefficient, further proving that the digital economy influences rural revitalization in China by enhancing human capital, thereby supporting Hypothesis 3.

## Conclusions and policy recommendations

### Discussion and conclusions

First, the digital economy index system’s construction level is biased. Using the dimensions of Internet-based and digital financial inclusion as proxy variables for evaluating the digital economy, researchers have examined the effects of the digital economy on China’s rural revitalization [59]. Despite falling within its purview, the Internet and digital finance differ from the digital economy. Studying the effects of the digital economy on rural revitalization can produce biased findings if the aforementioned two dimensions are used to assess the digital economy and factors such as the industrialization of the digital economy and the digitization of industries are ignored. Instead, This study uses data from 30 provinces and cities in China from 2012 to 2020 to empirically investigate the three characteristics of digital economy infrastructure, industrial digitization, and digital industrialization are combined to develop an index system for evaluating the digital economy. The findings show that the digital economy has significantly contributed to the revitalization of China’s countryside. Consequently, previous research findings are improved, and a more thorough and scientific assessment index system is built for assessing the digital economy.

Second, research on how the digital economy is revitalizing China’s rural areas at the new urbanization level has been limited, with the finding differing significantly due to variances in impact. Some studies have revealed that new urbanization is a significant element influencing rural revitalization in China [60]; however, they have ignored the role of the digital economy in this process. The promotion effect of the digital economy on rural revitalization differs for different characteristics. As a result, this study examines how the digital economy, with varying levels of new urbanization, affects rural rejuvenation in China. The findings imply that the digital economy has a bigger impact on China’s rural regeneration in places with higher rates of new urbanization.

Third, Prior research has mostly examined how China’s rural revitalization is impacted by the digital economy, generally assessing this influence from a single angle [61]. Which will lead to biased research findings.The first mediating factor is STI. Because research and development (R&D) activities are frequently characterized by uncertainty, high failure rates, and lengthy R&D cycles, the STI output is more representative and persuasive in measuring the effect of STI. Therefore, this study approaches the topic from the perspective of STI output. Published studies have quantified STI using the number of patent applications, which are products of scientific research, but this number overlooks the economic benefit indicator of agricultural technology market turnover. This study used two indicators to reflect the scientific research output and economic benefits of rural STI to more thoroughly measure the level of STI in rural areas, namely, the number of patent applications for new varieties of agricultural plants and the turnover of the agricultural technology market.This study applies the mediation model, and the empirical results show that STI is one essential mechanism through which the digital economy affects rural revitalization in China.

Moreover, the elderly and young people who remain in rural areas are too young to take on the task of revitalizing their communities due to the significant departure of young people observed in recent years. As a result, sufficient rural human capital is required to support rural revival projects. The human capital found in rural labor forces is more critical to China’s efforts to revitalize their communities. However, previous studies have mainly used the average years of schooling of the rural population to measure rural human capital [62]. Therefore, this paper uses the real human capital per laborer to describe the rural human capital status. The rural work force’s per capita human capital is an all-encompassing measure that includes aspects such as health, education, and hands-on training. Drawing more thorough and rigorous study results is possible by using this indicator, which can more closely reflect the real state of human capital in rural areas. Using a mediated effects model, this paper confirms that human capital plays a significant role in the transmission mechanism through which the digital economy influences China’s rural revival.

### Policy recommendations

First, expenditure on digital infrastructure should be increased, and the digital economy should be encouraged in various fields. As the digital economy promotes the development of rural revitalization, the government should invest more in digital infrastructure in rural areas to ensure the growth of the digital economy and take full advantage of its benefits, subsequently enabling the creation of a digital countryside. This would significantly increase the comprehensive effect and benefits of the digital economy in rural industries.

Second, multiple initiatives should be implemented to reduce the disparities in regional digital economy development. The promotion of China’s digital economy for rural revitalization is weaker in the central and western areas, suggesting that digital development should be accelerated in these regions. Therefore, the governments in these regions should make efforts in pivotal areas, such as creating a digital talent strategy, cultivating digital technology, and developing digital businesses, to form a new industry driven by data resources. Additionally, they should accelerate crossregional cooperation, such as in the “East Digital and West Calculation” project, to establish a spatial pattern for the development of the digital economy and for the widespread effects of digital technology, particularly in technology innovation and application, to address the issue of uneven development.

Third, science, technology, human capital, and innovation should be enhanced in rural areas to expedite regeneration. Through innovation in science, technology, and human resources, the digital economy brings an endogenous force to China’s rural revitalization development. Furthermore, it is necessary to improve the innovation system, increase innovation investment, establish innovation platforms, and gather professional talents to improve STI levels. These actions can surpass the corresponding “critical value” and offer scientific and technological support for the digital economy to foster the development of rural revitalization. Moreover, human capital development should be prioritized. By controlling rural market management, enhancing rural resource allocation methods, and reforming talent appraisal and use mechanisms, a pattern of beneficial interaction between rural talent resources and development can be established.

### Research limitations and outlook

Nonetheless, this study has many shortcomings that should be addressed in subsequent research. First, the lack of sufficient data prevented the examination of digital platforms and sharing economies within the digital economy. Subsequent studies can include these data. Second, due to space limitations, the spatial correlation of digital economy development in each province was not considered. Thus, future research can be conducted on the spatial spillover effect of digital economy development. Third, the digital economy’s impact on rural revitalization was evaluated based on relatively limited factors. Consequently, the validity and impartiality of the findings should be improved in further research.

## Supporting information

S1 File (DOC)
