# Correction: Can the digital economy contribute to rural revitalization? A case of from China?

**DOI:** 10.1371/journal.pone.0320390

**Published:** 2025-03-10

**Authors:** Yuanquan Lu, Yuan Meng, Li Chen, Yuan Zhang

There are grammar errors in the article title. The correct title is: Can the digital economy contribute to rural revitalization? A case from China. The correct citation is: Lu Y, Meng Y, Chen L, Zhang Y (2024) Can the digital economy contribute to rural revitalization? A case from China PLOS ONE 19(10): e0310313. https://doi.org/10.1371/journal.pone.0310313.

There is an error in affiliation 2 for author Yuan Meng. The correct affiliation 2 is: School of Politics, Law and Management, Yan’an University, Shaanxi Province, China.
