# Supplementary material for: Can the digital economy contribute to rural revitalization? A case of from China?
Source: PLoS One. 2024 Oct 7;19(10):e0310313. doi: 10.1371/journal.pone.0310313 (PMC11457991; doi:10.1371/journal.pone.0310313)
Supplement: S1 File — (DOC) [file pone.0310313.s001.doc]

Appendix

Appendix A

**Entropy method**

There are typically subjective and objective assignment methods utilized when creating a multidimensional comprehensive evaluation index system. Compared to the objective way of assignment, the subjective method is more subjective and arbitrary. The weights of the complete assessment index system for rural revitalization are therefore calculated using the entropy technique in this research. Before calculating the entropy weight technique, all of the indicators in the above rural revitalization index system must be standardized in this work to eliminate the quantitative relationship between the indicators and make them comparable. Consequently, the following is the standardizing formula for this paper: Formulae standard:

*Positive indicators:*
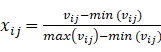
(1)

*Negative indicators:*
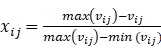
(2)

1≤
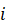
≤
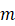
;1≤
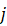
≤
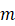


Equation (1) or equation (2) reflects the standardization of each variable, where
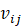
 represents the value of the jth indicator of the ith evaluation region, max(
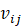
) and min(
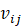
) represent the maximum and minimum values of the
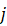
 indicator of the
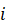
 evaluation region in turn, and
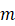
 represents the number of evaluated regions. number.

Using the entropy value technique, each variable must determine the weight of each indicator based on the criterion. Specific calculation stages for the entropy value technique include: Step 1: Through standardization
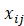
, Calculate the weight of the
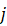
 the indicator value of the
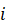
 evaluation area
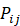
:


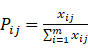
 (3)

Step 2: Calculate the entropy method for the j indicator
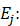


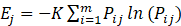
*;K=*
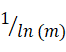
(4)

Step 3: Calculate the information value coefficient of the
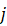
 indicator
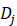


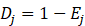
 (5)

Step 4: Calculate the weight of the entropy method for the
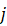
 indicator:


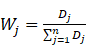
 (6)

Step 5: Calculate the composite score of the j indicator:


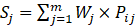
 (7)

Appendix B

New Urbanization indicator system, definitions and weights

| **Indicator Dimensions** | **Indicator Name** | **Average weight** | **Attributes** |  |
| --- | --- | --- | --- | --- |
| Economic Urbanization | Urban disposable income per capita | 0.2121 | + |  |
|  | Share of secondary and tertiary industries in GDP | 0.0412 | + |  |
| Population urbanization | Proportion of resident urban population | 0.1253 | + |  |
|  | Urban unemployment rate | 0.0857 | + |  |
| Social urbanization | Urban road area per capita | 0.0438 | + |  |
|  | Number of beds in urban medical institutions per 1,000 people | 0.0876 | + |  |
| Ecological urbanization | Green space per capita in cities | 0.0620 | + |  |
|  | Environmentally sound treatment of municipal domestic waste | 0.0316 |  |  |
| Cultural Urbanization | Percentage of urban population with high school education | 0.1444 | + |  |
|  | Urban residents' expenditure on education, culture and entertainment | 0.1663 | + |  |
